# Bio-guided isolation of anti-leishmanial natural products from *Diospyros gracilescens* L. (Ebenaceae)

**DOI:** 10.1186/s12906-021-03279-1

**Published:** 2021-03-31

**Authors:** Cyrille Armel N. Njanpa, Steven Collins N. Wouamba, Lauve Rachel T. Yamthe, Darline Dize, Brice Mariscal T. Tchatat, Patrick Valère F. Tsouh, Michel Nguiam Pouofo, Jean Bosco Jouda, Bruno Lenta Ndjakou, Norbert Sewald, Simeon Fogue Kouam, Fabrice Fekam Boyom

**Affiliations:** 1 Antimicrobial and Biocontrol Agents Unit, Laboratory for Phytobiochemistry and Medicinal Plants Studies, Department of Biochemistry, Faculty of science University of Yaounde I, P. O Box 812, Yaounde, Cameroon; 2 Department of Chemistry, Higher Teacher Training College, University of Yaounde I, P. O. Box 47, Yaounde, Cameroon; 3 Institute of Medical Research and Medicinal Plants Studies (IMPM), Ministry of Scientific Research and Innovation, P.O. Box 6133, Yaounde, Cameroon; 4 Department of Biochemistry, Faculty of science University of Bamenda, Bambili, P. O Box. 39, Bamenda, Cameroon; 5 Laboratory of Animal Physiology, Department of Animal Biology and Physiology, Faculty of Science, University of Yaounde I, P. O Box 812, Yaounde, Cameroon; 6 Organic and Bioorganic Chemistry, Faculty of Chemistry, University of Bielefeld, D-33501 Bielefeld, Germany; 7 Chemical Engineering and Mineral Industries School, University of Ngaoundere, P. O. Box 454, Ngaoundere, Cameroon

**Keywords:** *Diospyros gracilescens*, Ebenaceae, Hexane fraction, Isolated compounds, Antileishmanial, Cytotoxicity, 1-deoxyinositol

## Abstract

**Background:**

Plants represent an intricate and innovative source for the discovery of novel therapeutic remedies for the management of infectious diseases. The current study aimed at discovering new inhibitors of *Leishmania* spp., using anti-leishmanial activity-guided investigation approach of extracts from *Diospyros gracilescens* Gürke (1911) (Ebenaceae), targeting the extracellular (promastigotes) and intracellular (amastigotes) forms of *Leishmania donovani*.

**Methods:**

The plant extracts were prepared by maceration using H_2_0: EtOH (30:70, v/v) and further fractionated using a bio-guided approach. Different concentrations of *D. gracilescens* extracts, fractions and isolated compounds were tested in triplicate against *L. donovani* promastigotes and amastigotes in vitro. The antileishmanial potency and cytotoxicity on RAW 264.7 cells were determined using the resazurin colorimetric assay. The time kill kinetic profile of the most active sample was also investigated. The structures of all compounds were elucidated on the basis of extensive spectroscopic analyses, including 1D and 2D NMR, and HR-ESI-MS and by comparison of their data with those reported in the literature.

**Results:**

The hydroethanolic crude extract of *D. gracilescens* trunk showed the most potent antileishmanial activity (IC_50_ = 5.84 μg/mL). Further fractionation of this extract led to four (4) fractions of which, the hexane fraction showed the most potent activity (IC_50_ = 0.79 μg/mL), and seven (07) compounds that exhibited moderate potency (IC_50_ = 13.69–241.71 μM) against *L. donovani*. Compound 1-deoxyinositol (7) inhibited the promastigote and amastigote forms of *L. donovani* with IC_50_ values of 241.71 μM and 120 μM respectively and also showed the highest selectivity against *L. donovani* promastigotes (SI > 5.04). To the best of our knowledge, the antileishmanial activity of this compound is being reported here for the first time. The promising hexane fraction showed significant inhibition of parasites growth at different concentrations, but with no evidence of cidal effect over an exposure period of 120 h.

**Conclusions:**

The results obtained indicated that the hydroethanolic extract from the *D. gracilescens* trunk and the derived hexane fraction have very potent inhibitory effect on cultivated promastigotes and amastigotes of *L. donovani* parasite. The isolated compounds showed a lesser extent of potency and selectivity. However, further structure-activity-relationship studies of 1-deoxyinositol could lead to more potent and selective hit derivatives of interest for detailed drug discovery program against visceral leishmaniasis.

**Supplementary Information:**

The online version contains supplementary material available at 10.1186/s12906-021-03279-1.

## Background

Leishmaniasis is a severe, widespread zoonotic and parasitic disease caused by an intracellular flagellate protozoan of the genus *Leishmania*. The disease is generally transmitted between man and animals during a blood meal by the phlebotome female sandfly. About 20 different *Leishmania* species including *L. donovani* have been discovered to be pathogenic to human. The clinical features of the disease include a wide range of manifestations, including skin ulcers at the site of the infection or dissemination in visceral organs followed by anemia, leucopenia, fever and weakness [1, 2]. The World Health Organization estimates that 1.3 million new cases of leishmaniasis occur every year with 20,000 to 30,000 deaths annually [3]. Therefore, a great concern has been expressed by the WHO, as leishmaniases are considered as neglected tropical diseases [4]. Visceral leishmaniasis (VL), caused by *L. donovani* is the most dangerous form of the disease that can be lethal in human when untreated. It is considered as a serious public health problem worldwide, and especially in Africa where its significant morbidity and mortality require more effective chemotherapy [5]. Current available chemotherapy includes the first line treatment drugs such as pentavalent antimonials, meglumine antimoniate (glucantime) and sodium stibogluconate (pentostam) and second line drugs such as amphotericin B, pentamidine, paromomycin and miltefosine [6, 7]. However, these drugs are limited by factors such as emergence of drug resistance, especially with the pentavalent antimonials and challenges of toxicity, short half-life and high cost of drugs, as well as failure of patient to comply with treatment [8, 9]. Due to the limitations of current chemotherapeutic regimes and in the absence of effective and sustainable vaccines, there is a persistent need for alternative and readily available sources for treatment of leishmaniasis. In this respect, natural products offer good sources for new drug discovery [10].

This paper describes the in vitro antileishmanial activity of natural products from *D. gracilescens*, a plant of Ebenaceae family. It is a forest tree widely distributed in West and Centre regions of Cameroon. Furthermore, there is no mention of use of *D. gracilescens* in traditional medicine in Cameroon. However, related species such as *D. bipindensis* (Gürke), *D. conocarpa* (Gürke & K. Schum.) and *D. malabarica* ((Descr.) Kostel.) are widely used by Baka Pygmies for the treatment of malaria, sleeping sickness and respiratory disorders [11]. Globally, *Diospyros* spp. are known above all, as fishing poisons, especially in South East Asia and in the Philippines. They are also widely-used medications in traditional African medicine, mainly against leprosy. The roots are used as purgative in the Central African Republic, against pneumonia in Zimbabwe and schistosomiasis in Malawi [12, 13]. The first chemical study of *D. gracilescens* led to the isolation of few compounds such as: lupeol, betulin, betulinic acid, isodiospyrin (II) and sitosterol [14]. Of note, this is the first report of antileishmanial guided isolation of the chemical constituents of *D. gracilescens*.

## Methods

### Phytochemical investigation of D. gracilescens

#### Collection of plant material and preparation of the crude extracts

The plant materials (root, trunk, stem bark and leaf) of *D. gracilescens*, were collected in March 2017 at Eloumdem mountain (GPS coordinates: Latitude 3°49′00″N, Longitude 11°25′60″E), in the Centre Region of Cameroon and were identified by comparison with a voucher specimen (No. 2016 / SRFK) from the National Herbarium of Cameroon by Mr. Victor Nana, a botanist.

The collected plant materials were dried under shelter at room temperature and further ground to obtain the powders. The tinctures were prepared by maceration of 4000 g of each powder in aqueous ethanol 70% (15 L, 48 h × 3). The resulting macerates were filtered using Whatman filter paper No. 2 and the filtrates concentrated on a Büchi rotary evaporator (Büchi Labortechnik AG - Flawil, Switzerland) under reduced pressure at 45–55 °C and further lyophilized using a freeze-dryer Alpha 2–4 LD plus (Christ, Germany) to yield the crude extracts. Each extract was kept dried in tightly stoppered bottles at 4 °C until it was used for the biological screenings.

#### Liquid-liquid partition of the trunk crude extract

**Fig. 1:**
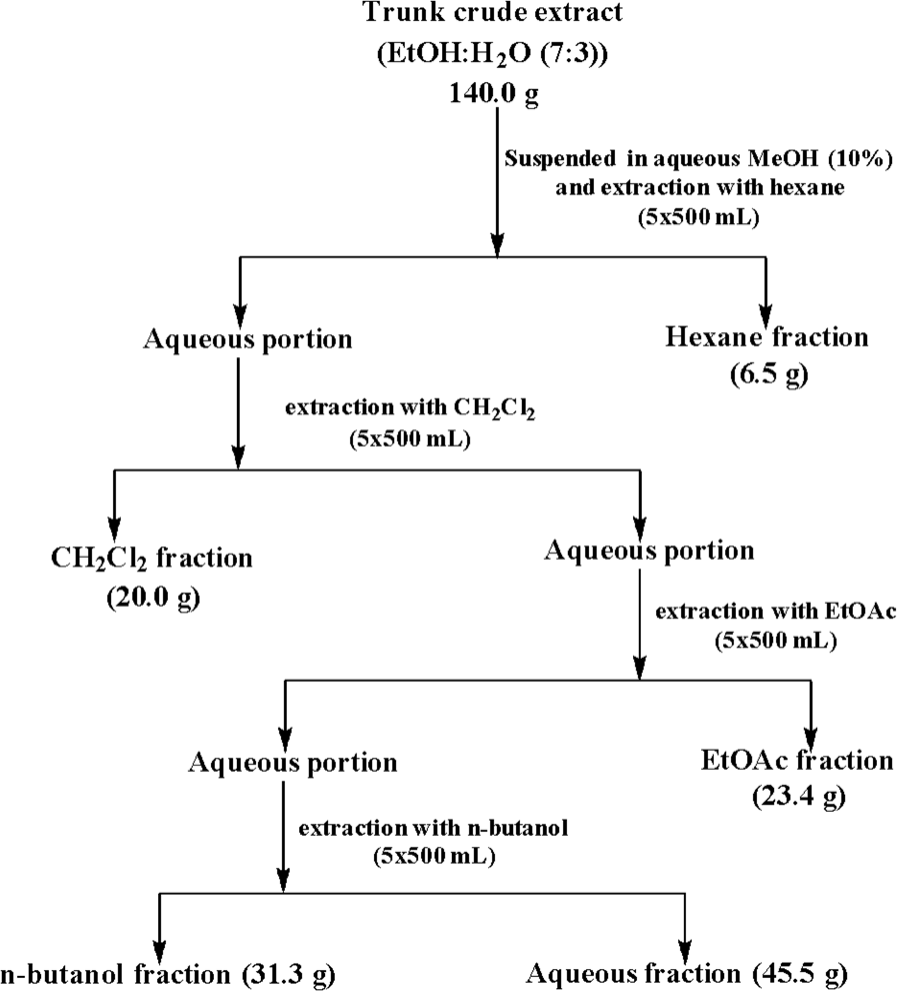
Fractionation procedure of the crude extract from the trunk of *D. gracilescens*. The crude extract suspended in water was sequentially partitioned using organic solvents

The crude extract of the trunk (150 g) which showed the best leishmanicidal activity was fractionated by liquid-liquid partition as shown in Fig. 1, according to the procedure described by Xie et al. [15]. Briefly, 140 g of the crude extract was suspended in water and then extracted with n-hexane, dichloromethane, ethyl acetate and *n*-butanol successively (Fig. 1). Each fraction was evaporated under reduced pressure at 45–55 °C and then the aqueous fraction was lyophilized. Five residues were obtained and respectively named fraction A from *n*-hexane [6.5 g, 4.6% yield], fraction B from dichloromethane [20.0 g, 14.3% yield], fraction C from ethyl acetate [23.4 g, 16.7% yield], fraction D from *n*-butanol [31.3 g, 22.35% yield] and fraction E for the remaining aqueous residue (45.5 g, 32.50% yield). Each of the afforded fractions was submitted to antileishmanial screening and the promising ones (fractions A, B and D) having IC_50_ values below 2 μg/mL were selected and submitted to chromatographic separation.

#### Chromatography of the active fractions

##### Chromatography of fraction A

A portion of *n*-hexane fraction (m = 6 g; IC_50_ = 0.78 μg/mL) was subjected to silica gel column chromatography eluting with an isocratic system of *n*-hexane/ethyl acetate (95/5). Three hundred sub-fractions of ca 100 mL each were collected and combined into 10 sub-fractions A1—A10 based on their TLC profiles. Sub-fractions A1 to A6 consisted of a mixture of fatty acids and were not investigated further. Lupeol (1) (1.11 mg) and a mixture of *β*-sitosterol (4) and stigmasterol (5) (14.8 mg) were obtained from sub-fractions A7–8 (n-hexane/EtOAc (95/5), 1.5 g) and sub-fractions A9&10 (*n*-hexane/EtOAc 90/10, 2.2 g and 80/20, 22 mg) respectively [16, 17].

##### Chromatography of fraction B

The dichloromethane fraction (m = 19 g; IC_50_ = 1.63 μg/mL) was subjected to column chromatography on 230–400 mesh silica gel (Merck, Darmstadt, Germany) eluting with a gradient of ethyl acetate in *n*-hexane. Ninety sub-fractions of ca 250 mL each were collected and combined into 4 major sub-fractions B1—B4 according to their TLC profiles. Sub-fraction B1 (*n*-hexane/EtOAc (95/5), 8.1 g) was found to contain mainly fatty substances and traces of hexane soluble compounds. Sub-fraction B2 (n-hexane/EtOAc (90/10), 2.1 g) was subjected to repeated silica gel column chromatography using a mixture of *n*-hexane:ethyl acetate in a gradient mode as eluent to afford the mixture of *β*-sitosterol (4) and stigmasterol (5) (17.6 mg). Sub-fraction B4 (*n*-hexane/EtOAc (65/35), 4.5 g) was fractionated on silica gel column chromatography with an isocratic solvent system of n-hexane/ethyl acetate (30/70) to afford a mixture of compounds 2 and 3 which were further purified using a sephadex LH-20 (Sigma-Aldrich, Munich, Germany) column chromatography with a mixture of CH_2_Cl_2_/MeOH (20/80) as eluent to afford pure betulin (2) (2.0 mg) and betulinic acid (3) (27 mg) [17–19]. Sub-fraction B3 was not further studied due to limited quantity.

##### Chromatography of fraction D

The n-butanol fraction (m = 30 g; IC_50_ = 1.11 μg/mL) was subjected to silica-gel column chromatography eluting with a mixture of *n*-hexane/acetone/methanol in a gradient mode. Twenty sub-fractions of ca 400 mL each were collected and combined into 5 major sub-fractions D1—D5 based on their TLC profiles. Beta-Sitosterol glucoside (6) (185 mg) precipitated in sub-fraction D1 (*n*-hexane/Acetone 30/70). Compound 1-deoxyinositol (7) (30 mg) precipitated in sub-fraction D5 (*n*-hexane/Acetone/Methanol (3/6/1)) and was filtered before being purified by recrystallization in acetone/water (90/10) [11, 20]. Data on sub-fractions D2–3 are not presented in this paper because they are under further scrutiny.

#### HPLC–DAD–ESI-MS analysis of extracts from D. gracilescens

##### Sample preparation

Each extract was dissolved in HPLC grade methanol at a concentration of 0.5 mg/mL, then filtrated through a syringe-filter-membrane. Each aliquot obtained (5 μL) was injected into the UPLC–DAD-HRESI/MS Dionex Ultimate 3000 HPLC (Germany) apparatus used to perform the analyses.

##### HPLC-MS conditions

High resolution mass spectra were obtained with an OTOF Spectrometer (Bruker, Germany) equipped with a HRESI source and a UV–vis absorbance detector. The spectrometer was operated in positive mode (mass range: 100–1500, with a scan rate of 1.00 Hz) with automatic gain control to provide high-accuracy mass measurements with 2 ppm deviation using Na Formate as calibrant. Mass spectra were simultaneously acquired using electrospray ionization in the positive ionization mode. The following parameters were used for experiments: spray voltage of 4.5 kV, capillary temperature of 200 °C. Nitrogen was used as sheath gas (10 l/min). The spectrometer was attached to an Ultimate 3000 (Thermo Fisher, USA) HPLC system consisting of LC-pump, UV traces were measured at 215, 218, 254, 280 and 330 nm and UV spectra—Diode Array Detector— (DAD) was recorded between 190 and 600 nm, auto sampler (injection volume 5 μl) and column oven (35 °C). The separations were performed using a Synergi MAX-RP 100A (50 × 2 mm, 2.5 μ particle size) with a H_2_O (+ 0.1% HCOOH) (A)/acetonitrile (+ 0.1% HCOOH) (B) gradient (flow rate 500 μL/min). Samples were analyzed using a gradient program as follows: 95% A isocratic for 1.5 min, linear gradient to 100% B over 6 min, after 100% B isocratic for 2 min, the system returned to its initial condition (90% A) within 1 min, and was equilibrated for 1 min.

##### Identification of peaks

Identification of all constituents was performed by UPLC–DAD-HRESI/MS analysis and by comparing the UV, MS spectra and MS/MS fragmentation of the selected peaks in the sample chromatogram with those of data reported the literature of SciFinder database.

### Screening of extracts for biological activity

#### Parasite culture and maintenance

The cryopreserved promastigote form of *L. donovani* (1S (MHOM/SD/62/1S) was obtained from Bei Resources (https://www.beiresources.org/) and is routinely cultured at the Antimicrobial and Biocontrol Agents Unit, University of Yaoundé I, in Medium 199 (Sigma, Darmstadt, Germany) supplemented with 10% Heat-Inactivated fetal Bovine Serum (HIFBS) (Sigma, Darmstadt, Germany) and 100 IU/mL penicillin and 100 μg/mL streptomycin. The culture was maintained in 75 Cm^2^ cell culture flask at 28 °C and checked for growth daily and sub-cultured everyday 72 h [21].

#### Determination of the antileishmanial activity of plant extracts and fractions

##### Inhibitory assay against *L. donovani* promastigotes

The antileishmanial activity of *D. gracilescens* crude extracts, derived fractions and compounds against cultured *L. donovani* promastigotes was evaluated using the resazurin colorimetric assay as described by Siqueira-Neto et al. [22]. The stock solutions were prepared by dissolving each sample in 100% dimethyl sulfoxide (DMSO) and subsequently diluted serially in non-supplemented culture medium. To assess the antileishmanial activity, 4 × 10^5^ promastigotes/mL/well were seeded in a 96 well microtiter plate and treated with 5-fold diluted concentrations of *D. gracilescens* extracts (0.16, 0.8, 4, 20 and 100 μg/mL) for 72 h at 28 °C. The viability rate of promastigotes positively correlated with the amount of pink resorufin that was produced through the reduction of blue resazurin by the dehydrogenase enzymes in the inner mitochondrial membrane of the living parasites. Briefly, promastigotes from a logarithmic phase culture (4 × 10^5^ cells/mL; 90 μL) were seeded in 96-well microtiter plates and were treated with 10 μl of inhibitors at different triplicate concentrations ranging 100 μg/mL-0.16 μg/mL for extracts and fractions and 50 μg/mL-0.08 μg/mL for compounds. The final concentration of DMSO in each well was not higher than 1%. Plates were incubated for 28 h at 28 °C, followed by the addition of 1 mg/mL resazurin (Sigma, Darmstadt, Germany). The negative and positive controls were 0.1% DMSO and amphotericin B (Sigma, Darmstadt, Germany) (10–0.016 μg/mL) respectively. After an additional incubation for 44 h, plates were then read on a Magelan Infinite M200 fluorescence multi-well plate reader (Tecan, Männedorf, Switzerland) at an excitation and emission wave lengths of 530 and 590 nm respectively. For each sample, growth percentages were calculated and dose–response curves were constructed to determine the 50% inhibitory concentration (IC_50_) using the GraphPad Prism-version 5.0 software (San Diego, California, USA).

##### Inhibitory assay against *L. donovani* amastigotes

The effect of plant isolates against the intracellular amastigote form of *L. donovani* was evaluated essentially as described by Jain et al. [23] with some modifications. Briefly, macrophage Raw 264.7 cells (4 × 10^3^ cells/well) were seeded in 96 well plates and incubated for 6 h at 37 °C under 5% CO_2_ for adhesion of the cells. Afterwards, the non-adherent cells were washed-out with sterile PBS. The adherent Raw 264.7 cells were infected with metacyclic promastigotes (4 × 10^5^ cells) at an infection ratio of 1:10 macrophage: parasites and incubated for 24 h at 37 °C under 5% CO_2_ in order to allow infection of macrophages by metacyclic promastigotes. Thereafter, the overlying medium was removed, and the monolayer cells with internalized amastigotes were carefully washed four times with PBS to remove free parasites. Freshly prepared M199 medium containing 10% FBS and the test extracts were added in triplicate to the infected cells at serially diluted concentrations and incubated for 48 h at 37 °C under 5% CO_2_. After incubation, 0.05% Sodium dodecyl sulfate (SDS) was added in each well for 30 s for controlled lysis followed by M199 with 10% FBS as macrophage lysis stopper. Resazurin reagent (250 μg/mL) was thereafter added in each well and the plates were incubated for 24 h followed by fluorescence recording at λexcitation = 530 nm and λemission = 590 nm using a Tecan Infinite M200 microplate reader (Tecan). Inhibition percentages were calculated using Microsoft Excel Software and median inhibitory concentration (IC_50_) obtained from dose-response curves using GraphPad Prism 5.0. Software.

#### Cytotoxicity assay

The cytotoxicity profile of extracts, fractions and compounds was assessed using the Alamar blue assay [24] against Raw 264.7 cells duly cultivated in complete Dulbecco’s Modified Eagle’s Medium (DMEM) containing 13.5 g/L DMEM (Sigma Aldrich), 10% fetal Bovine Serum (Sigma Aldrich), 0.2% sodium bicarbonate (w/v) (Sigma, Darmstadt, Germany) and 50 μg/mL gentamycin (Sigma Aldrich). Globally, macrophages were seeded into 96-wells cell-culture flat-bottomed plates at a density of 10^4^ cells in final volume of 100 μL of complete medium/well and incubated for 24 h at 37 °C, 5% CO_2_, 70% RH in a ICO 105 memmert incubator (memmert, Schwabach, Germany) to allow cell adhesion. Ten μL of each serially diluted test sample were added in triplicate wells and assay plates were then incubated for 48 h in the same experimental conditions. Growth control consisted of 0.1%DMSO (100% growth) and positive control of podophyllotoxin (Sigma, Darmstadt, Germany) at 20 μM. Cell proliferation was checked by adding 10 μL of a stock solution of resazurin (0.15 mg/mL in sterile PBS) to each well followed by plates incubation during 4 h. Fluorescence was then read on a Tecan Infinite M200 fluorescence multi-well plate reader at an excitation/emission of 530/590 nm. Results were expressed as 50% cytotoxic concentrations (CC_50_). Selectivity indices (CC_50_/IC_50_, defining the balance between cytotoxicity and antileishmanial activity) were calculated for each test substance.

#### Concentration/time inhibition kinetics of the most active fraction

The growth inhibitory effect of the most active fraction against *L. donovani* promastigotes was examined by culturing parasites (4 × 10^5^ cells/ml) in freshly prepared complete M199 medium in the presence and absence of varying concentrations of the hexane fraction (1/2 IC_50_, IC_50_, 2x IC_50_, 4x IC_50_), using amphotericin B as positive control (1/2 IC_50_, IC_50_, 2x IC_50_, 4x IC_50_) for 120 h. The number of viable parasites was determined after every 24 h over 120 h by staining with trypan blue. Quantification of viable parasites was achieved by counting the parasites with clear cytoplasm (non-stained) using a Neubauer hemocytometer with cover slips. Three independent experiments were performed for each sample.

### Data analysis

All the activity data represent mean ± standard deviation (SD) from three independent experiments. Microsoft Excel Software was used to calculate the percentage of inhibition. The IC_50_ and CC_50_ values were determined using GraphPad Prism 5.0 Software with data fitted by non-linear regression.

### Statistical analysis

Data were expressed as mean ± SEM (standard error of mean). Statistical analysis was performed by one-way ANOVA (analysis of variance) followed by the Bonferroni post-test using GraphPad 7 software. Difference was considered as significant at *p* < 0.05.

## Results

### Phytochemical analysis data

**Fig. 2:**
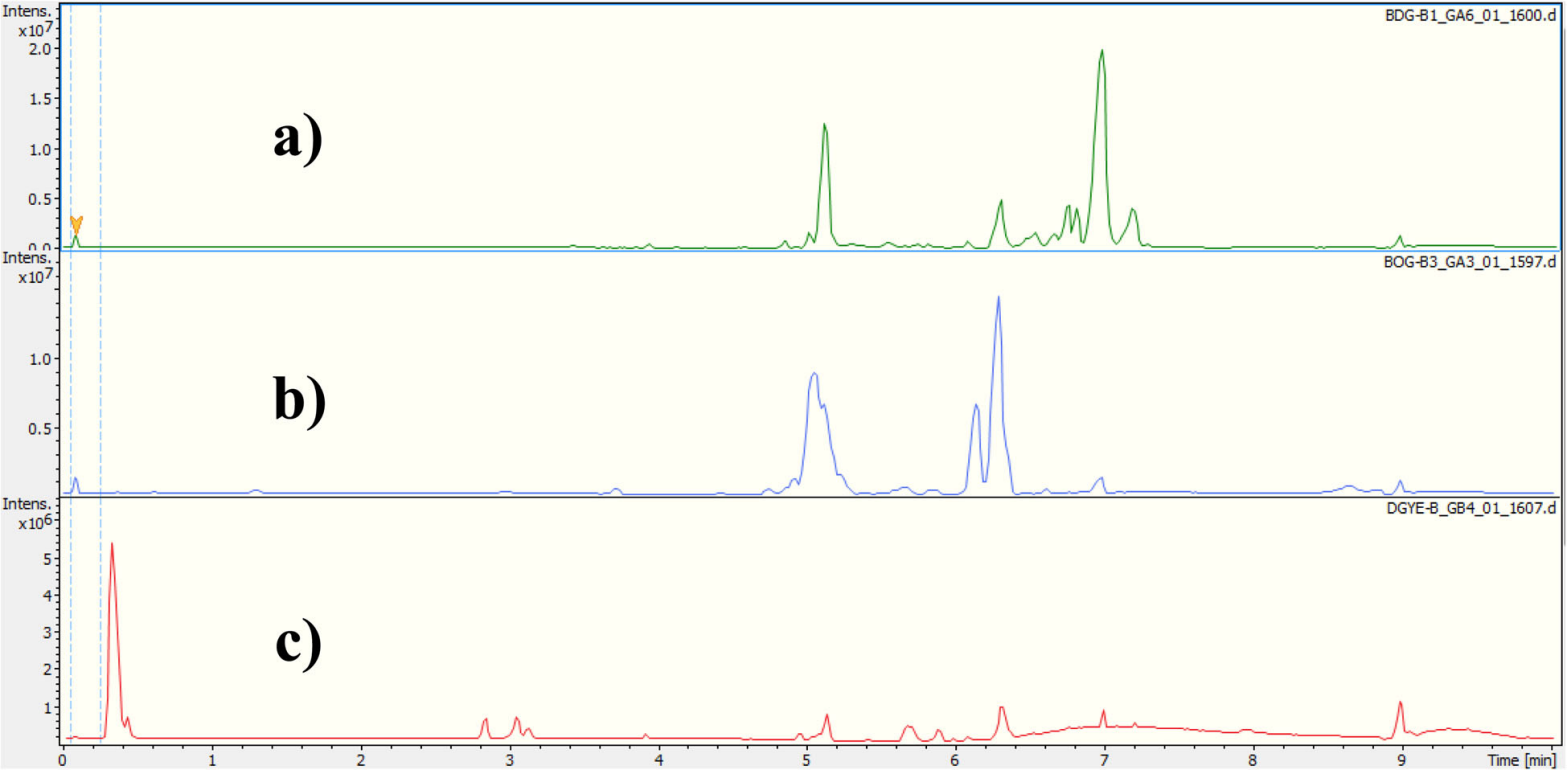
HPLC profiles of the crude extract and fractions from the trunk of *D. gracilescens* (TIC: m/z 150–1000). a crude extract; b Dichloromethane fraction; c Hexane fraction

The crude extract, the hexane and dichloromethane soluble fractions of the trunk of *D. gracilescens* were analyzed by UPLC coupled to both diode array and mass spectrometry detectors. The latter was used with an electrospray ionization (ESI) source in positive ion mode. A representative base peak chromatogram and all ions MS (Fig. 1) indicating that the used UPLC conditions allowed a good separation of a large percentage of compounds. The compounds were recognizable from their characteristic UV spectra, which were identified based on the UPLC–DAD–HRESI-MS data and subsequent confirmation by comparison with literature data. The chromatographic profile and spectroscopic data are presented in Table 1 and Fig. 2 below.

**Table 1:** FT-MS product ions of detected compounds in the trunk extract of *D. gracilescens*

N°	RT (min)	[M + H]^+^		UV, λ_max_ (nm)	molecular formula	Name of compound
		Exp.	Calcd.			
1	7.19	663.4740	663.4772	222	C_46_H_62_O_3_	Chlorobiumquinone
2	6.99	431.3689	431.3672	222	C_32_H_46_	NI
3	6.82	483.3621	483.3621	450	C_30_H_46_O	NI
4	6.77	427.3946	427.3946	222	C_30_H_50_O	Lupeol
5	6.65	469.3465	469.3421	222	C_32_H_46_O	NI
6	6.57	479.3887	479.3884	222	C_33_H_50_O_2_	NI
7	6.21	391.3003	391.2995	222	C_28_H_38_O	NI
8	5.13	579.3116	579.3105	218	C_38_H_42_O_5_	NI

*NI* Not Identified

### Biological activities data

#### Antileishmanial activity of plant samples

The inhibitory potential of *D. gracilescens* extracts against *L. donovani* promastigotes was measured by direct counting of live promastigotes after parasite exposure to various concentrations of extract. The IC_50_ values of the different crude extracts are shown in Table 2 below.

**Table 2:** Anti-leishmanial activity of *D. gracilescens* crude extracts against promastigotes of *L. donovani*

Plant	Part	Solvent	Promastigote of *L. donovani* (MHOM/SD/62/1S) IC_50_ ± SD (μg/ml)
*D. grascilisens*	Root	H_2_0: EtOH	> 100
	Stem bark	H_2_0: EtOH	> 100
	Leaf	H_2_0: EtOH	> 100
	Trunk	H_2_0: EtOH	5.84 ± 0.20
Positive control	Amphotericin B		0.34 ± 0.22

Activity data are expressed as mean ± Standard deviation (SD) from triplicate experiments; IC_50_: 50% Inhibitory Concentration

The results shown in Table 2 indicate that only the extract from the trunk of *D. grascilisens* exerted antileishmanial activity with an IC_50_ value of 5.84 μg/mL. The other extracts were non-active up to 100 μg/mL. The trunk extract was therefore progressed for bio-guided investigation.

#### Bio-guided fractionation of the trunk extract of D. grascilisens

Fractionation of the trunk extract was performed by liquid-liquid partition to afford four (04) main fractions that were tested for activity against the promastigotes and amastigotes forms of *L. donovani* (Table 3).

**Table 3:** Anti-leishmanial activity and selectivity Indexes of fractions from the trunk crude extract against promastigotes and amastigotes of *L. donovani*

Extract/ fraction	IC_50_ (Promastigotes) (μg/ml ± SD)	IC_50_ (Amastigotes) (μg/ml) ± SD	Raw267.4 CC_50_ (μg/ml ± SD)	SI (Promastigotes)	SI (Amastigotes)
Trunk crude extract	5.84 ± 0.20^f^	35.69 ± 0.26^f^	> 100	> 18.56	> 2.80
Hexane fraction (A)	0.79 ± 0.09^b^	8.06 ± 0.39^b^	> 100	> 126.74	> 12.40
Dichloromethane fraction (B)	1.63 ± 0.11^d^	10.97 ± 0.11^c^	> 100	> 61.16	> 9.11
Ethyl-acetate fraction (C)	2.36 ± 0.06^e^	16.05 ± 0.12^d^	> 100	> 42.30	> 6.23
n-butanol fraction (D)	1.11 ± 0.10^c^	22.08 ± 0.09^e^	> 100	> 89.60	> 4.53
Water residue (E)	> 100	–	–	–	–
Positive control	0.34 ± 0.22^a^	0.12 ± 0.11^a^	/	/	/

Activity data are mean ± Standard deviation (SD) from triplicate experiments; IC_50_: 50% Inhibitory Concentration; CC_50_: 50% Cytotoxic Concentration; SI: Selectivity Index. Along each column, IC_50_ values with the same letter superscripts are not significantly different, Bonferroni test (*p* > 0.05)

Results from Table 3 indicate that bio-guided fractionation of the crude trunk extract (IC_50_ = 5.84 μg/mL) has resulted in more potent fractions, with increase in activity in the range of 7.4–2-fold against *L. donovani* promastigotes (IC_50_ = 0.79–2.36 μg/mL). The hexane fraction showed the highest potency (IC_50_ = 0.79 μg/mL), followed respectively by the n-butanol (IC_50_ = 1.11 μg/mL), dichloromethane (IC_50_ = 1.63 μg/mL) and ethyl acetate (IC_50_ = 2.36 μg/mL) fractions. The water residue showed no activity up to 100 μg/mL. Activity data against *L. donovani* amastigotes indicated decreased potency of the crude trunk extract (IC_50_ = 35.69 μg/mL) and the hexane fraction (IC_50_ = 8.06 μg/mL). Overall, the extract and fractions showed greater selectivity against *L. donovani* promastigotes (18 < SI < 127) than against the amastigotes (2.8 < SI < 12.4). The hexane fraction exhibited the highest selectivity against *L. donovani* promastigotes (SI > 126.7) and amastigotes (SI > 12.4).

**Fig. 3:**
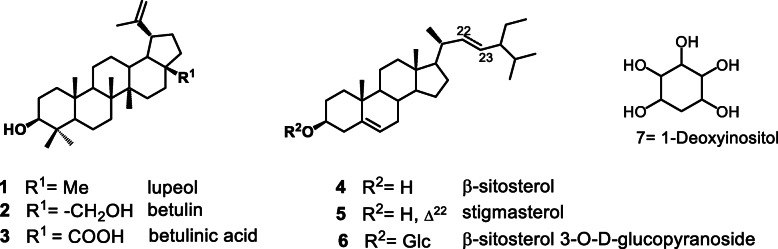
Isolated compounds from *D. gracilescens.* Structures of the isolated compounds were elucidated by means of physical and spectroscopic methods

Further fractionation of fraction A led to the isolation of lupeol (1) and a mixture of sterols (4). Fraction B led to betulin (2) and betulinic acid (3) and fraction D led to *β*-sitosterol glucoside (6) and 1-deoxyinositol (7) as shown in Fig. 3 below. The structures of these compounds were elucidated on the basis of spectroscopic analyses, including 1D and 2D NMR, and HR-ESI-MS and by comparison of their data with those reported in the literature (see supplementary information). These compounds were also tested for activity against the promastigote and amastigote forms of *L. donovani*. The results achieved are presented in Table 4.

**Table 4:** Anti-leishmanial activity and selectivity indexes of isolated compounds against *L. donovani* promastigotes and amastigotes

Compound	IC_50_ (Promastigotes) (μM ± SD)	IC_50_ (Amastigotes) (μM ± SD)	Raw267.4 CC_50_ (μM ± SD)	SI (Promastigotes)	SI (Amastigotes)
Lupeol (1) from fraction A	23.22 ± 0.08	46.66 ± 0.65	19.28 ± 0.3	0.82	0.41
Betulin (2) from fraction B	NT	NT	ND	ND	ND
Betulinic acid (3) from fraction B	13.69 ± 0.07	18.46 ± 0.01	82.39 ± 0.09	6.02	4.46
Mixture of sterols (4) from fraction A	46.83 ± 0.20	17.83 ± 0.17	18.26 ± 0.18	0.39	1.02
β-sitosterol glucoside (6) from fraction D	49.34 ± 0.12	32 ± 0.22	28.60 ± 0.07	0.58	0.89
1-deoxyinositol (7) from fraction D	241.71 ± 0.16	120 ± 0.18	> 1218 ± 0.13	> 5.04	> 10.15
Amphotericin B	0.37 ± 0.22	0.13 ± 0.11		/	/

Activity data are expressed as mean ± Standard deviation (SD) from triplicate experiments; IC_50_: 50% Inhibitory Concentration, ND: Non-Determined; NT: Non-Tested (Betulin not tested due to insufficient quantity)

Globally, the isolated compounds exhibited moderate inhibition of *L. donovani* promastigotes (IC_50_: 13.69–241.71 μM) and amastigotes (17.83–120 μM). Of note, the activity of 1-deoxyinositol (IC_50_ of 241.71 μM and 120 μM against promastigotes and amastigotes respectively) is being reported here for the first time.

#### Kinetics of parasite killing as a relation to time and inhibitor concentration

**Fig. 4:**
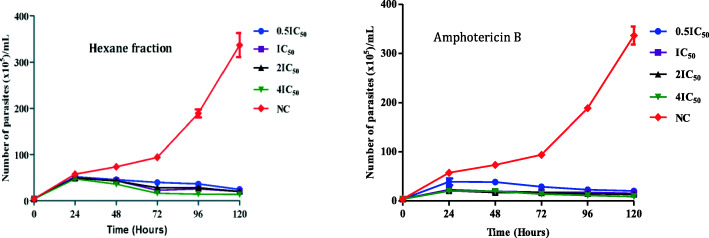
Kill kinetics of *L. donovani promastigotes* in relation to drug concentration and time. Cells were treated with different concentrations of (a) hexane fraction and (b) amphotericin B. The growth kinetic curves were plotted as *number of viable parasites* vs *time* with data collected every 24 h over a period of 120 h; NC = Negative control; Each data point represents mean ± SD from three experiments

The following graphs below (Fig. 4) show the time kill kinetic of the most active (Hexane) fraction (A), compared to the positive control, amphotericin B (B).

The ability of the hexane fraction to fast-kill *L. donovani* promastigotes was assessed at different concentrations points (0.5x IC_50_, IC_50_, 2x IC_50_ and 4x IC_50_) relative to untreated parasites culture over a period of 120 h. The results showed that treatment with increasing concentrations of the hexane fraction and amphotericin B resulted in a significant reduction in promastigote replication after 24 h (Fig. 4). In the meantime, an exponential growth was observed in untreated parasite culture.

At all tested concentrations of hexane fraction and amphotericin B, a regular reduction of the viability of treated parasite cultures was observed up to 120 h, with however no evidence of cidal effect.

## Discussion

The investigation of extracts from *D. grascilesens* for antileishmanial activity identified the hydroethanolic trunk extract as a promising starting point for bio-guided study. This is the first report describing the antileishmanial activity of extracts from *D. grascilesens*. However, extracts from other *Diospyros* spp. have been previously investigated in this direction. Of note, Lenta et al. [25] demonstrated the capacity of the dichloromethane-methanol (1:1) extract of *D. canaliculata* (De Wild*.*) to inhibit the growth of axenic amastigotes of *L. donovani*. On another hand, Dhar et al. [26] reported that ethanolic extracts of *D. montana* (Roxb) and *D. peregrina* ((Gaertn.) Gürke) possess antiprotozoal activity against *Entamoeba histolytica*, antiviral activity against Ranikhet disease virus and hypoglycemic activities in albino rats. Rocío et al. [27] also demonstrated the in vitro antimycobacterial potency of the stem bark extract from *D. anisandra* (S.F. Blake) against a resistant strain of *M. tuberculosis*. In other studies, Hazra et al. [28] demonstrated the anti-tumour activity of bark extract from *D. ferrea* ((Willd.) Bakh). Asolkar et al. [29] showed the antibacterial activity of leaf and seed extracts from *D. montana* and Satish and Sunil [30] demonstrated the anti-diabetic and antioxidant potential of the ethanolic bark extract of *D. malabarica*.

Based on the criteria set for antileishmanial activity of plant extracts by Camacho et al. [31], the promising hydroethanolic trunk extract of *D. grascilesens* was further fractionated yielding the hexanic fraction as the more active and selective against the extracellular and the intracellular forms of *L. donovani* parasite. Further fractionation of this fraction led to six (6) compounds identified as lupeol, betulin, betulinic acid, mixture of sterols, β-sitosterol glucoside and 1-deoxyinositol. Among these compounds, betulinic acid showed the most potent activity and selectivity against both promastigote and amastigote forms of *L. donovani*. There are few studies in the literature reporting the activity of betulin and betulinic acid and derivatives against *Leishmania* parasites. Indeed, similarly to our findings, Sousa et al. [32] have reported moderate antileishmanial activity (23-55 μM) of semisynthetic lupane triterpenoids, betulin and betulinic acid when tested alone, and their synergistic effects with miltefosine, an alkylphosphocholine drug with demonstrated activity against various parasite species (including *Leishmania infantum* parasites and amoeba) and cancer cells as well as some pathogenic bacteria and fungi [33]. Alakurtti et al. [34] also determined the activity of heterocyclic betulin derivatives on *L. donovani* amastigotes and the in vitro activity of betulin and betulinic acid derivatives against *L. donovani* amastigotes and promastigotes of *L. amazonensis*. Dominguez et al. [35] also reported the activity of betulinic acid acetate and betulinic acid methyl ester against promastigotes of *L. amazonensis*.

Betulinic acid has been already reported in the literature to possess a wide range of biological and medicinal properties, including anti-human immunodeficiency virus (HIV), antibacterial, antimalarial, anti-inflammatory, anthelmintic, antinociceptive, anti-herpes simplex viruses-1 (HSV-1), immune-modulatory, antiangiogenic, and anticancer activities [36, 37]. The activity of betulinic acid and its derivatives against the erythrocytic stage of the chloroquine-sensitive 3D7 *Plasmodium falciparum* strain was previously reported, as well as moderate antileishmanial activity on different *Leishmania* spp. [34, 38, 39]. Cassio et al. [40] also showed that the semi-synthetic derivatives of betulinic acid were able to prevent the parasite development and invasion into host cells, that are crucial events for *Trypanosoma cruzi* infection establishment, with potency similar to benznidazole.

In this study, lupeol was found to be active against promastigotes and moderately active against amastigotes of *L. donovani*, corroborating the previous findings. Indeed, the antileishmanial activity of lupeol against both promastigotes and amastigotes of *L. donovani* has been demonstrated in the literature [41]. Other previous studies indicated that lupeol isolated from aerial parts of *Vernonia scorpioides* displayed a weak antileishmanial activity [42, 43]. Also, studies have highlighted the activity of lupeol from the latex of *E. resinifera* and *E. officinarum* against promastigote of *L. infantum* [44]. Other studies attempting to establish the mechanism of action of lupeol were conducted by Ramos et al. [45] and showed that this compound mediates an increased cytoplasmic membrane depolarization which may promote enhanced cell membrane damage. They also suggested that the leishmanicidal activity could lead to disruption of the cytoplasmic membrane of *L. donovani* promastigotes as evidenced by DISC3 mediated fluorometric analysis. Whereas lupeol might mediate reduction in intracellular parasitic load was found to be executed through the induction of pro-inflammatory cytokine response and generation of Nitrite Oxide (NO) in *L. donovani* infected macrophages [41].

Betulinic acid and lupeol that showed the most potent activity in this study belong to the class of terpenoids. In fact, a number of terpenes are reputed to possess antileishmanial activity. Different authors suggested that the antileishmanial activity of these compounds could be related to the inhibition of proteins and nucleic acids synthesis or of a membrane-associated calcium-dependent ATPase pump [43, 46]. Indeed, previous studies have suggested that lipophilic compounds, such as triterpenes, act by a peculiar mechanism. These compounds can pass easily through the cytoplasmic membranes, affecting structures of their different layers of polysaccharides, fatty acids, and phospholipids, thus making them permeable [47]. Once they cross the membrane, the coagulation of cytoplasm can occur. These events are able to promote the interruption of specific metabolic pathways of lipids and proteins [48], interference in cell division [49, 50], or stimulate the depolarization of the mitochondrial membranes, which can lead the cell to trigger necrosis or apoptosis mechanisms [51].

Among the isolated compounds, 1-deoxyinositol exerted a promising activity against both promastigote and amastigote forms of *L. donovani*. This compound was previously detected by GC-MS in a moderately active (with IC_50_ value of 126.4 μg/mL) methanolic extract from the aerial part of *Scutellaria havanensis* against *L. amazonensis* promastigotes [52]. The antileishmanial activity of 1-deoxyinositol adds to the novelty of this work given that, to the extent of our knowledge, no previous report has been published on the antileishmanial activity of this compound. More interestingly, 1-deoxyinositol showed the highest selectivity against *L. donovani* promastigotes (SI > 5.04) as well as acceptable preference for amastigotes (SI > 10.15).

Overall, this study has indicated that the hexane fraction was 12 to 49-fold and 1.8 to 2.4-fold more active than the derived lupeol and mixture of sterols against the promastigote and amastigote forms of *L. donovani* respectively*.* Also, selectivity indexes greater than 152 and 11 for promastigotes and amastigotes respectively were obtained compared to the derived components. The activity profile of the hexane fraction portents a very probable synergistic interaction between its non-polar components to increase activity and selectivity (safety profile). The implication of these findings is of high significance in the use of *D. grascilesens* plant in traditional medicine to treat neglected tropical diseases (NTDs).

## Conclusion

This study reports the first detailed investigation aiming at determining the antileishmanial activity of natural products from *D. gracilescens* using a bio-guided approach. The hydroethanolic extract of the trunk showed promising profile, (IC_50_ = 5.84 μg/mL) and its bio-guided fractionation led to the most potent hexane fraction (IC_50_ = 0.79 μg/mL). Further fractionation of this fraction led to six compounds that also exhibited antileishmanial potency. The promising hexane fraction and derived active compounds represent potential raw materials for detail-oriented drug discovery against visceral leishmaniasis that exacts a very heavy toll to poor patients in remote endemic settings in Africa and elsewhere. Among the isolated compounds, 1-deoxyinositol has shown acceptable profile for further structure-activity-relationship studies in drug discovery program to unveil hits or leads adhering to the criteria defined earlier against visceral leishmaniasis. Of particular note is the activity profile of the hexane fraction that exerted the greatest potency and selectivity. It is a promising candidate for the development of a phytodrug against leishmaniasis.

## Supplementary Information

**Additional file 1.**

## Data Availability

All data generated or analyzed during this study are included in this published article and its Additional files.

## Abbreviations

CC_50_: 50% cytotoxic concentration
*D.*: *Diospyros*
DMEM: Dulbecco’s Modified Eagle’s Medium
DMSO: Dimethylsulfoxide
ESI: Electrospray Ionization
FBS: Fetal Bovine Serum
FT-MS: Fourier Transform Mass Spectrometry
HIFBS: Heat-Inactivated fetal Bovine Serum
HIV: Human Immunodeficiency Virus
HPLC: High Performance Liquid Chromatography
HRESI: High Resolution Electrospray Ionization
HSV-1: Herpes simplex viruses-1
IC_50_: 50% inhibitory concentration
L.: *Leishmania*
M199: Medium 199
M. tuberculosis: *Mycobacterium tuberculosis*
MS: Mass Spectrometry
NC: Negative control
NI: Not Identified
NO: Nitrite Oxide
NT: Non-tested
NTD: Neglected Tropical Disease
PBS: Phosphate Buffer Solution
SD: Standard deviation
SDS: Sodium Dodecyl sulfate
SI: Selectivity Index
*T. cruzi*: *Trypanosoma cruzi*
TIC: Total Ion Chromatogram
UPLC: Ultra Performance Liquid Chromatography
VL: Visceral Leishmaniasis
WHO: World Health Organization
