# Supplementary material for: Bio-guided isolation of anti-leishmanial natural products from Diospyros gracilescens L. (Ebenaceae)
Source: BMC Complement Med Ther. 2021 Mar 31;21:106. doi: 10.1186/s12906-021-03279-1 (PMC8011081; doi:10.1186/s12906-021-03279-1)
Supplement: Supplementary file 1 — Additional file 1. [file 12906_2021_3279_MOESM1_ESM.doc]

**Supplementary Material**

**Bio-guided isolation of anti-leishmanial natural products from *Diospyros gracilescens* L. (Ebenaceae)**.

Cyrille Armel N. Njanpa^1^, Steven collins N. Wouamba^2^, Lauve Rachel T. Yamthe^1,3^, Darline Dize^1^, Brice Mariscal T. Tchatat^1^, Patrick Valère F. Tsouh^4^, Michel Nguiam Pouofo^5^, Jean Bosco Jouda^7^, Bruno Lenta Ndjakou^2^, Norbert Sewald^6^, Simeon Fogue Kouam^2^*, Fabrice Fekam Boyom^1^*.

^1^Antimicrobial and Biocontrol Agents Unit, Laboratory for Phytobiochemistry and Medicinal Plants Studies, Department of Biochemistry, Faculty of science University of Yaounde I, P.O Box 812, Yaounde, Cameroon.

^2^Department of Chemistry, Higher Teacher Training College, University of Yaounde I, P. O. Box 47, Yaounde, Cameroon.

^3^Institute of Medical Research and Medicinal Plants Studies (IMPM), Ministry of Scientific Research and Innovation, P.O. Box 6133, Yaounde, Cameroon.

^4^Department of Biochemistry, Faculty of science University of Bamenda, Bambili, P.O Box. 39, Bamenda, Cameroon.

^5^Laboratory of Animal Physiology, Department of Animal Biology and Physiology, Faculty of Science, University of Yaounde I, P.O Box 812, Yaounde, Cameroon.

^6^Organic and Bioorganic Chemistry, Faculty of Chemistry, University of Bielefeld, D-33501, Bielefeld, Germany.

^7^Chemical Engineering and Mineral Industries School, University of Ngaoundere, P. O. Box 454, Ngaoundere, Cameroon.

*Corresponding Author:

**Fabrice FEKAM BOYOM**

Antimicrobial and Biocontrol Agents Unit, Laboratory for Phytobiochemistry and Medicinal Plants Studies, Department of Biochemistry, Faculty of science, University of Yaounde I, P.O Box 812, Yaounde, Cameroon. Tel.: +237677276585; Email address: [fabrice.boyom@fulbrightmail.org](mailto:fabrice.boyom@fulbrightmail.org)

**Siméon FOGUE KOUAM**

Department of Chemistry, Higher Teacher Training College, University of Yaounde I, P. O. Box 47, Yaounde, Cameroon Tel.: +237694464535; Email address: [kfogue@yahoo.com](mailto:kfogue@yahoo.com)

**Abstract**

**Background:** Plants represent an intricate and innovative source for the discovery of novel therapeutic remedies for the management of infectious diseases. The current study aimed at discovering new inhibitors of *Leishmania* spp., using anti-leishmanial activity-guided investigation approach of extracts from *Diospyros gracilescens* (Ebenaceae), targeting the extracellular (promastigotes) and intracellular (amastigotes) forms of *Leishmania donovani*.

**Methods:** The plant extracts were prepared by maceration using H_2_0: EtOH (30:70, v/v) and further fractionated using a bio-guided approach. Different concentrations of *D.* *gracilescens* extracts, fractions and isolated compounds were tested against *Leishmania donovani* promastigotes and amastigotes *in vitro*. The antileishmanial potency and cytotoxicity on RAW 264.7 cells were determined using the resazurin colorimetric assay. The time kill kinetic profile of the most active sample was also investigated. The structures of all compounds were elucidated on the basis of extensive spectroscopic analyses, particularly 1D and 2D NMR, and HR-ESI-MS and by comparison of their data with those reported in the literature.

**Results:** The hydroethanolic crude extract of *D.* *gracilescens* trunk showed the most potent antileishmanial activity (IC_50_ = 5.84 µg/mL). Further fractionation of this extract led to four (4) fractions of which, the hexane fraction showed the most potent activity (IC_50_ = 0.79 µg/mL), and seven (07) compounds that also exhibited potency and selectivity (IC_50_ = 13.69-241.71 µM) against *L. donovani*. Interestingly, 1-deoxyinositol (**7**) showed promising antileishmanial activity against the promastigote and amastigote forms of *L. donovani* (IC_50_ values of 241.71 and 120 µM respectively) and also the highest selectivity against *L. donovani* promastigotes (SI> 5.04) as well as acceptable action preference for amastigotes (SI> 1.09). To the best of our knowledge, the antileishmanial activity of this compound is being reported here for the first time. The promising hexane fraction showed significant inhibition of parasites growth at different concentrations, but with no evidence of cidal effect over an exposure period of 120 hours.

**Conclusions:** The results obtained indicated that the hydroethanolic extract from the *D.* *gracilescens* trunk and the derived hexane fraction have very potent inhibitory effect on cultivated promastigotes and amastigotes of *Leishmania* *donovani* parasite. The isolated compounds showed a lesser extent of potency and selectivity. However, further structure-activity-relationship studies of 1-deoxyinositol could lead to more potent and selective hit derivatives of interest for detailed drug discovery program against visceral Leishmaniasis.

**Keywords:** *Diospyros* *gracilescens*, Ebenaceae**,** hexane fraction, isolated compounds, antileishmanial, cytotoxicity, 1-deoxyinositol.

**Spectroscopic data of compounds 1−7**


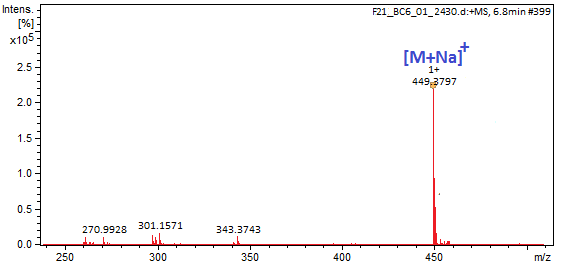


Figure S1: HRESI Mass spectrum of lupeol (**1**)

Figure S2: ^1^H NMR spectrum of lupeol (**1**) (CDCl_3_, 500 MHz)

Figure S3: ^13^C NMR spectrum of lupeol (**1**) (CDCl_3_, 125 MHz)

Figure S4: ^1^H NMR spectrum of betulin (**2**) (CDCl_3_, 500 MHz)

**
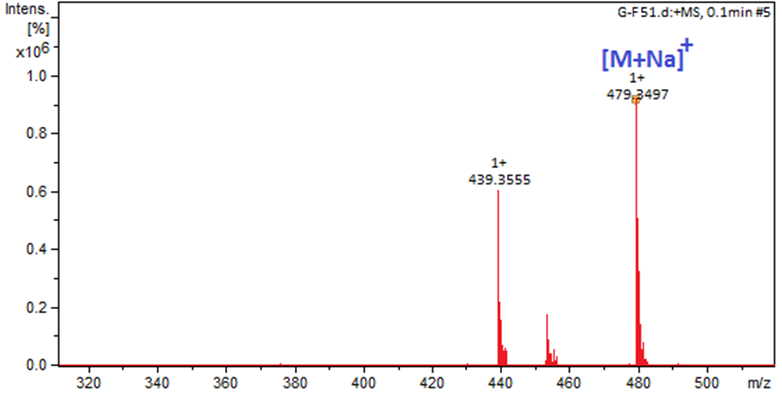
**

Figure S5: HRESI Mass spectrum of betulinic acid (**3**)

Figure S6: ^1^H NMR spectrum of betulinic acid (**3**) (CDCl_3_, 500 MHz)

Figure S7: ^13^C NMR spectrum of betulinic acid (**3**) (CDCl_3_,, 125 MHz)


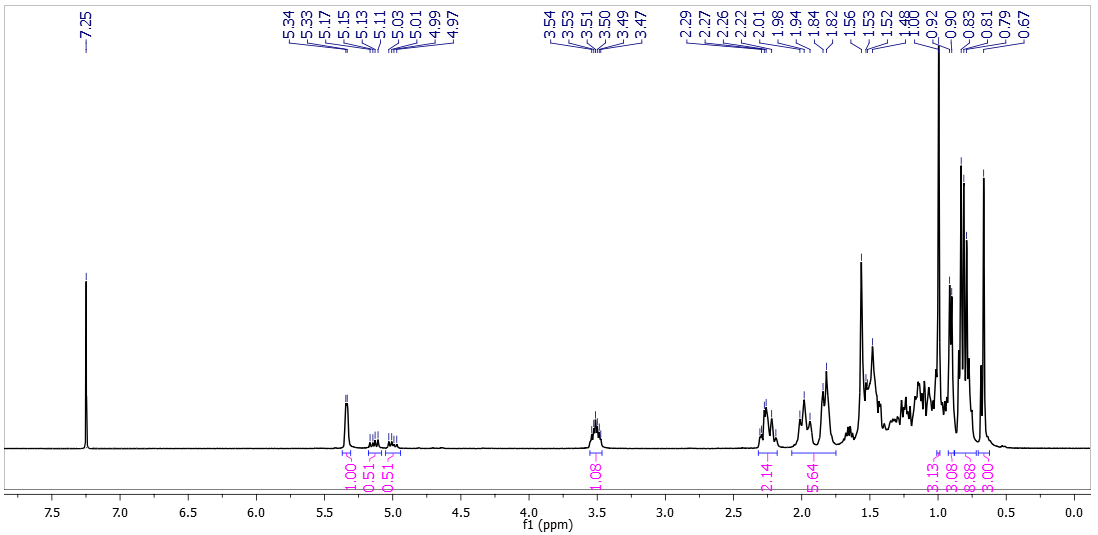


Figure S8: ^1^H NMR spectrum of a mixture of compounds **4** and **5** (CDCl_3_, 500 MHz)


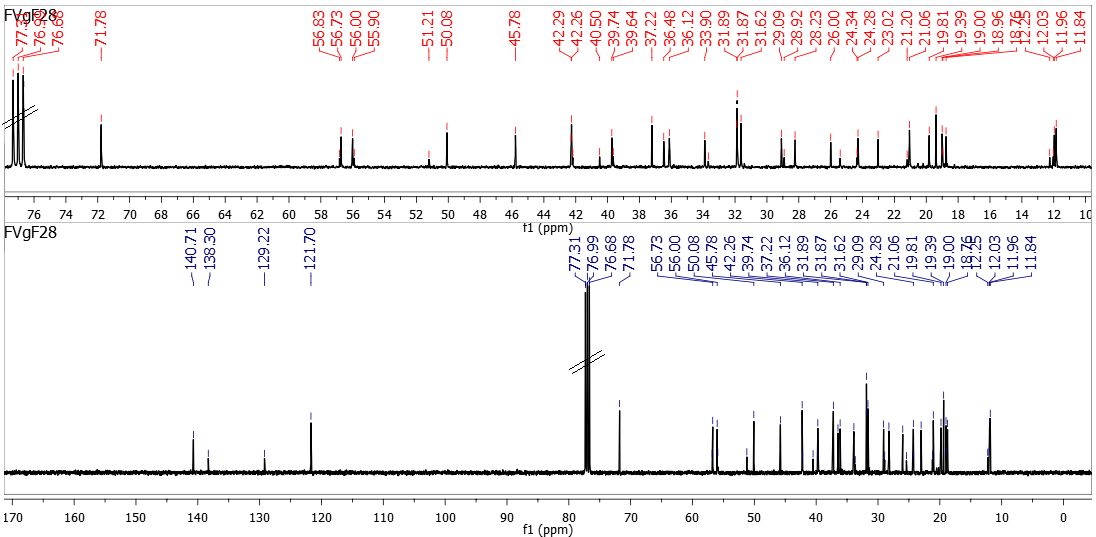


Figure S9: ^13^C NMR spectrum of a mixture of compounds **4** and **5** (CDCl_3_, 125 MHz)


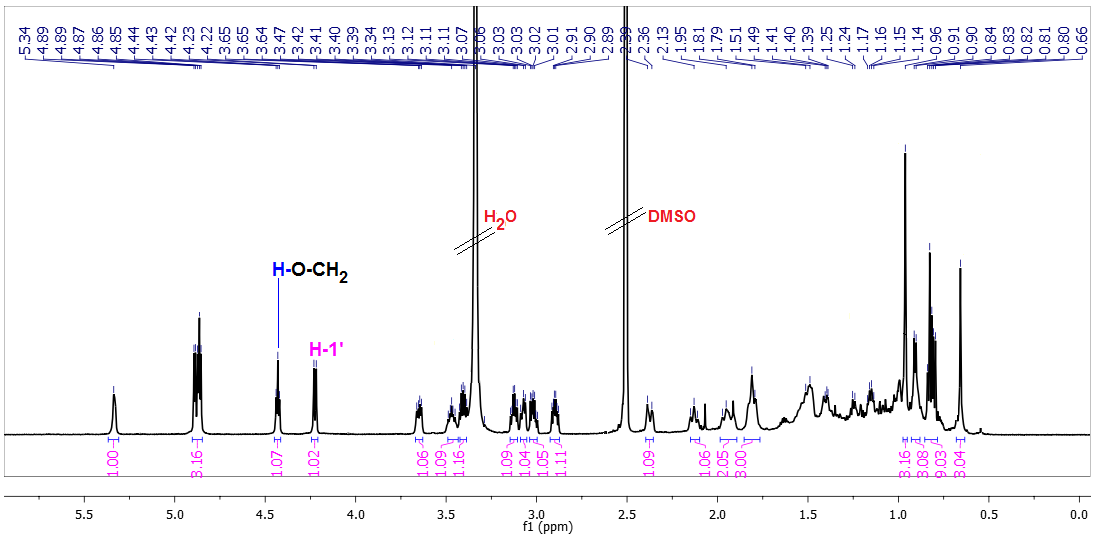


Figure S10: ^1^H NMR spectrum of *β*-sitosterol 3-O-D-glucopyranoside (6) (DMSO-*d_6_*, 500 MHz)


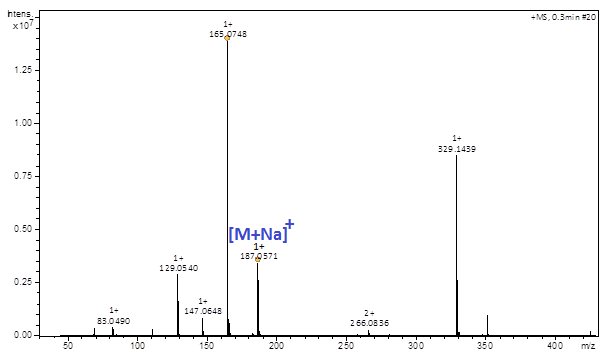


Figure S11: HRESI Mass spectrum of 1-deoxyinositol (**7**)


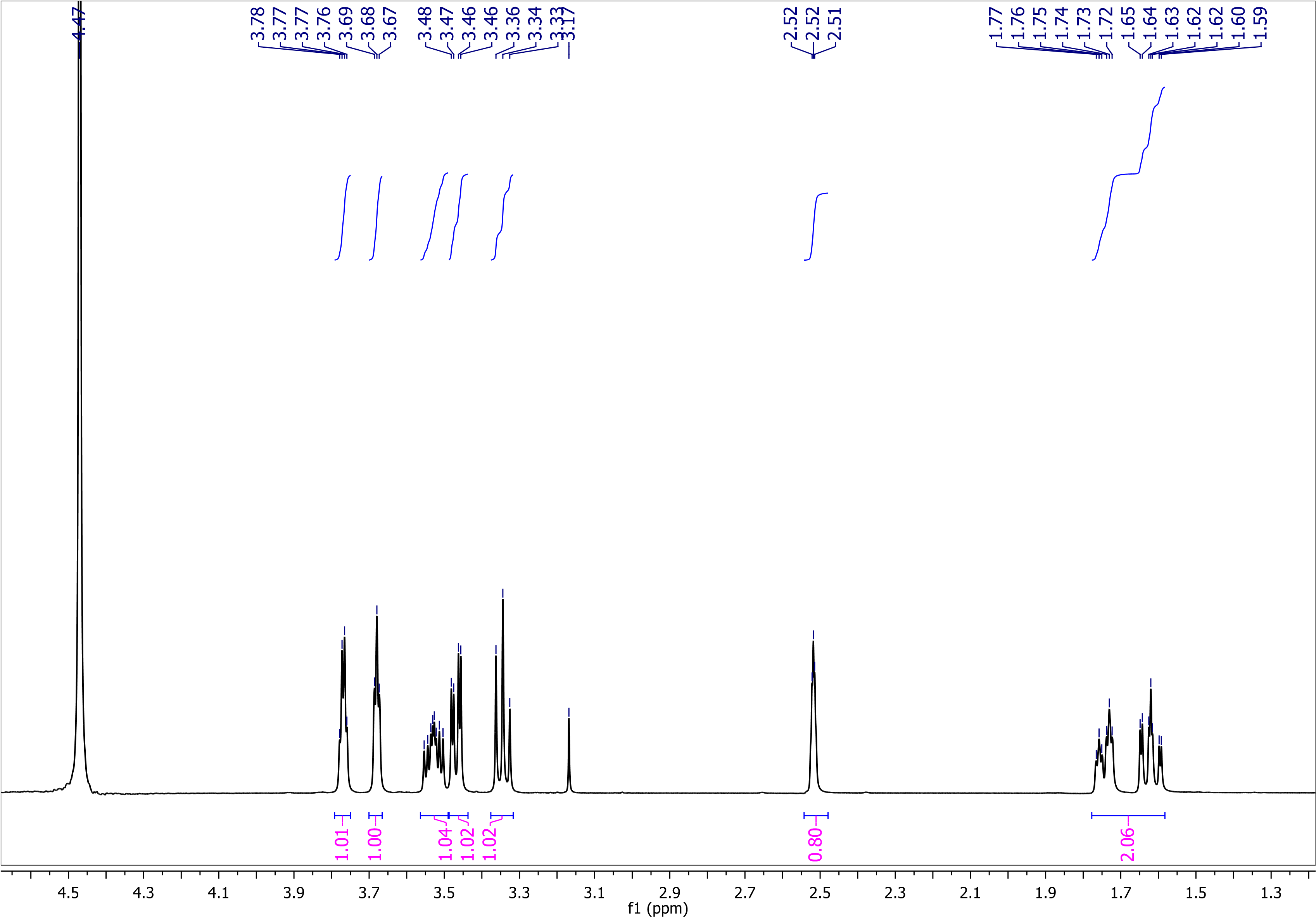


Figure S11: ^1^H NMR spectrum of 1-deoxyinositol (**7**) (DMSO-*d_6_*, 500 MHz)


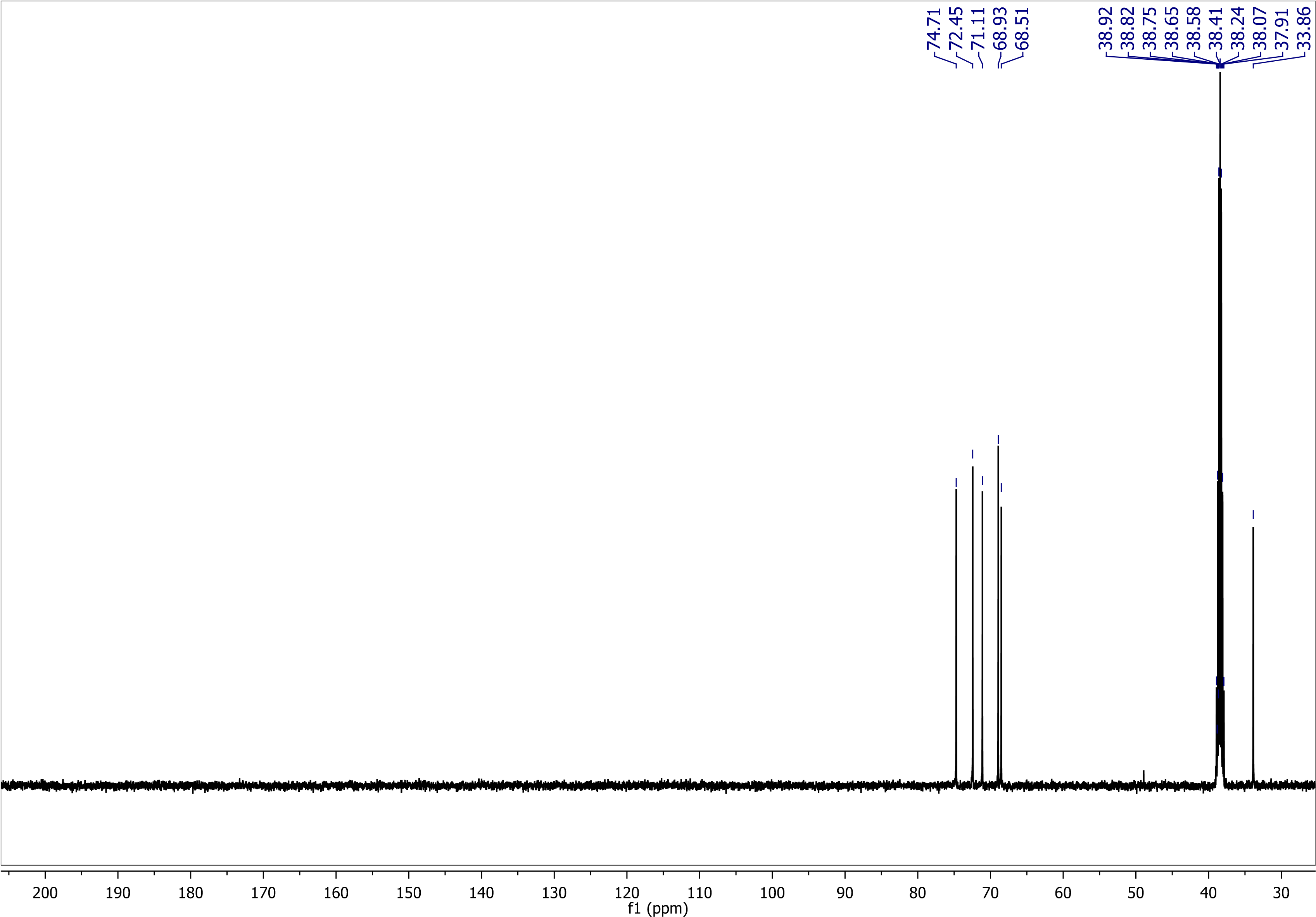


Figure S12: ^13^C NMR spectrum of 1-deoxyinositol (**7**) (DMSO-*d_6_*, 125 MHz)

**Table 1S:** Comparison of ^1^H-NMR data in CDCl_3_ of compounds **1**, **2** and **3** with lupeol, betulin and betulinic acid previously reported in the literature [**17**, **18**, **19**]

|  | Lupeol (**1**) (CDCl_3_) | | Betulin (**2**) (CDCl_3_) | | Betulinic acid (**3**) (CDCl_3_) | |
| --- | --- | --- | --- | --- | --- | --- |
| no. | *δ*_H_ (nH, *m*, *J(Hz)*) | | *δ*_H_ (nH, *m*, *J(Hz)*) | | *δ*_H_ (nH, *m*, *J(Hz)*) | |
|  | Ref. (400MHz) | Exp. (500MHz) | Ref. (400MHz) | Exp. (500MHz) | Ref. (500MHz) | Exp. (500MHz) |
| 3 | 3.20 (1H, *m*) | 3.22 (1H, *dd*, *J*=9.6, 6.2) | 3.18 (1H, *brd*, *J*=5.3) | 3.11 (1H, *st*, *J*=5.1) | 3.22 (1H, *dd*, *J*=11.5, 5.0) | 3.22 (1H, *dd*, *J*=11.4, 4.9) |
| 23 | 0.79 (3H, *s*) | 0.81 (3H, *s*) | 0.75 (3H, *s*) | 0.69 (3H, *s*) | 0.76 (3H, *s*) | 0.78 (3H, *s*) |
| 24 | 0.85 (3H, *s*) | 0.85 (3H, *s*) | 0.80 (3H, *s*) | 0.76 (3H, *s*) | 0.83 (3H, *s*) | 0.85 (3H, *s*) |
| 25 | 0.94 (3H, *s*) | 0.97 (3H, *s*) | 0.96 (3H, *s*) | 0.90 (3H, *s*) | 0.93 (3H, *s*) | 0.96 (3H, *s*) |
| 26 | 0.97 (3H, *s*) | 0.99 (3H, *s*) | 0.97 (3H, *s*) | 0.91 (3H, *s*) | 0.99 (3H, *s*) | 0.99 (3H, *s*) |
| 27 | 1.06 (3H, *s*) | 1.06 (3H, *s*) | 0.99 (3H, *s*) | 0.95 (3H, *s*) | 1.01 (3H, *s*) | 1.00 (3H, *s*) |
| 28 | 0.77 (3H, *s*) | 0.79 (3H, *s*) | 3.79 (1H, *d*, *J*=10.8)  3.33 (1H, *d*, *J*=10.8) | 3.73 (1H, *d*, *J*=10.6)  3.27 (1H, *d*, *J*=10.6) | — | — |
| 29 | 4.70 (1H, *d*, *J*=0.4)  4.55 (1H, *d*, *J*=0.4) | 4.71 (1H, *brs*)  4.59 (1H, *brs*) | 4.70 (1H, *d*, *J*=0.4)  4.55 (1H, *d*, *J*=0.4) | 4.71 (1H, *brs*)  4.59 (1H, *brs*) | 4.74 (1H, *brs*)  4.60 (1H, *brs*) | 4.76 (1H, *brs*)  4.63 (1H, *brs*) |
| 30 | 1.69 (3H, *brs*) | 1.71 (3H, *s*) | 1.69 (3H, *brs*) | 1.71 (3H, *s*) | 1.69 (3H, *s*) | 1.71 (3H, *s*) |
| Ref. = Literature data; Exp. = experimental data | | | | | | |

**Table 2S:** Comparison of ^1^H-NMR data in CDCl_3_ of mixture of compounds **4** /**5** with mixture of *β*-sitosterol/ stigmasterol and ^1^H-NMR data in DMSO-d6 of compound **6** with *β*-sitosterol 3-*O*-*D*-glucopyranoside previously reported in the literature [**16**, **19**]

|  | *β*-sitosterol/ stigmasterol (**4**and **5**) (CDCl_3_) | | *β*-sitosterol 3-O-D-glucopyranoside (**6**) (DMSO-d6) | |
| --- | --- | --- | --- | --- |
| no. | *δ*_H_ (*m*, *J(Hz)*) | | *δ*_H_ (*m*, *J(Hz)*) | |
|  | Ref. (500MHz) | Exp. (500MHz) | Ref. (500MHz) | Exp. (500MHz) |
| 3 | 3.53 (*dd*, *J*= 9.6, 4.8) | 3.52 (*dd*, *J*= 9.8, 5.0) | 3.42 (*dt*, *J* =11.8, 6.0) | 3.42 (*m*) |
| 6 | 5.34 (1H, *d*, *J*= 5.2) | 5.33 (1H, *d*, *J*= 5.5) | 5.35 (*d*, *J* =5.0) | 5.34 (*brd*, *J* =5.1) |
| 18 | 0.68 (s) | 0.67 (s) | 0.68 (*s*) | 0.66 (*s*) |
| 19 | 1.01 (s) | 1.00 (s) | 0.96 (*s*) | 0.96 (*s*) |
| 21 | 0.92 (*d*, *J*= 6.0) | 0.91 (*d*, *J*= 6.2) | 0.88 (*d*, *J* = 6.6) | 0.84 (*d*, *J* = 6.8) |
| 22 | 5.16 (*dd*, *J*= 15.0, 8.4) | 5.15 (*dd*, *J*= 15.3, 8.6) | 1.25 (*m*) | 1.24 (*m*) |
| 23 | 5.03 (*dd*, *J*= 15.0, 8.4) | 5.02 (*dd*, *J*= 15.3, 8.5) | 1.16 (*m*) | 1.15 (*m*) |
| 26 | 0.82 (*d*, *J*= 7.2) | 0.81 (*d*, *J*= 7.3) | 0.80 (*m*) | 0.80 (*m*) |
| 27 | 0.81 (*d*, *J*= 7.2) | 0.79 (*d*, *J*= 7.3) | 0.85 (*m*) | 0.83 (*m*) |
| 29 | 0.85 (*d*, *J*= 8.0) | 0.85 (*d*, *J*= 8.2) | 0.83 (*m*) | 0.81 (*m*) |
| 1′ | — | — | 4.22 (d, *J* =7.8) | 4.23 (d, *J* =7.6) |
| Ref. = Literature data; Exp. = experimental data | | | | |

**Table 3S:** Comparison of ^1^H and ^13^C -NMR data in DMSO-d6 of compound **7** with 1-Deoxyinositol previously reported in the literature [**20**]

|  | ^1^H (500MHz) and ^13^C(125MHz) -NMR of 1-deoxyinositol (**7**) in (DMSO-d6) | | | |
| --- | --- | --- | --- | --- |
| no. | Ref. | | Exp. | |
|  | *δ*_C_ | *δ*_H_ (nH, *m*, *J(Hz)*) | *δ*_C_ | *δ*_H_ (*m*, *J(Hz)*) |
| 1 | 69.0 | 3.42 (1H, *m*) | 68,9 | 3.47 (1H, *m*) |
| 2 | 74.6 | 3.27 (1H, *t*, *J*= 8.9) | 74.7 | 3.34 (1H, *t*, *J*= 9.0) |
| 3 | 71.1 | 3.38 (1H, *dd*, *J*= 3.0, 9.2) | 71.1 | 3.46 (1H, *dd*, *J*= 3.0, 9.0) |
| 4 | 72.3 | 3.58 (1H, *t,* *J*= 3.1) | 72.4 | 3.68 (1H, *t,* *J*= 3.0) |
| 5 | 68.6 | 3.67 (1H, *d*, *J*= 3,3) | 68.5 | 3.77 (1H, *m*) |
| 6 | 33.7 | 1.60 (2H, *m*) | 33.8 | 1.62 (2H, *ddd*, *J*= 13.0, 3.0, 0.7) |
| Ref. = Literature data; Exp. = experimental data | | | | |
